# A Systematic Review of CD34+ Stem Cell Therapy as an Innovative and Efficient Treatment for the Management of Refractory Angina

**DOI:** 10.7759/cureus.32665

**Published:** 2022-12-18

**Authors:** Vruti Vithani, Bansi Sutariya, Diana M Montenegro, Michael Chukwu, Paghunda Ehsan, Rawia N Aburumman, Shivani Ishwarya Muthanna, Swathi Radhakrishnan Menon, Sai Sri Penumetcha

**Affiliations:** 1 Internal Medicine, California Institute of Behavioral Neurosciences & Psychology, Fairfield, USA; 2 General Medicine, Government Medical College, Surat, IND; 3 General Surgery, California Institute of Behavioral Neurosciences & Psychology, Fairfield, USA; 4 General Surgery, Pilgrim Hospital, Boston, GBR; 5 Research, California Institute of Behavioral Neurosciences & Psychology, Fairfield, USA; 6 Internal Medicine, Hayatabad Medical Complex Peshawar, Peshawar, PAK; 7 Internal Medicine, Mu'tah University, Amman, JOR; 8 Internal Medicine, Vydehi Institute of Medical Sciences and Research Centre, Bengaluru, IND; 9 Pediatrics, California Institute of Behavioral Neurosciences & Psychology, Fairfield, USA; 10 General Medicine, California Institute of Behavioral Neurosciences & Psychology, Fairfield, USA; 11 General Medicine, Chalmeda Anand Rao Institute of Medical Sciences, Karimnagar, IND

**Keywords:** stem cell transplantation, cd34+ stem cell therapy, microvascular angina, angina pectoris, refractory angina

## Abstract

Despite optimal medical treatment, many individuals suffering from severe coronary artery disease are not suitable candidates for further revascularization. Therapeutic angiogenesis has attracted continuous interest to increase myocardial perfusion. Cell therapy using autologous stem cells expressing Cluster of Differentiation 34 plus (CD34+) offers a special therapeutic choice for individuals with refractory angina, seeing as CD34+ stem cells can restore microcirculation. We searched PubMed, PubMed Central (PMC), and Google Scholar to find the relevant articles to write this systematic review about the role of CD34+ stem cell therapy in the management of refractory angina. Additionally, we provided a brief explanation of CD34+ cells and their mechanism of action. Along with the positive finding of other trials, a recent open-label, single-center intracoronary CD34+ cell therapy for the treatment of coronary endothelial dysfunction in patients with angina and nonobstructive coronary arteries (IMPROvE-CED) clinical trial published in 2022 concluded improvement in coronary blood flow, a significant reduction in daily as-needed sublingual nitroglycerin use and improvement in Canadian Cardiovascular Society (CCS) angina class were observed after autologous CD34+ cell treatment. In conclusion, refractory angina management and overall prognosis may be revolutionized once this treatment is approved.

## Introduction and background

A growing number of people with significant coronary artery disease become poor candidates for further revascularization while receiving the best possible medical care and remaining symptomatic [1]. According to the European Society of Cardiology, refractory angina is “a chronic condition characterized by the presence of retrosternal chest pain caused by coronary insufficiency in the presence of coronary artery disease which cannot be controlled by a combination of medical therapy, angioplasty, and coronary bypass surgery” [2]. Patients with refractory angina (RA) have few treatment choices, which include antianginal medicine, enhanced external counterpulsation (EECP), risk factor reduction, and angioplasty with stenting [1]. There are unclear estimates of the prevalence of refractory angina, ranging from 300,000 to 1.7 million individuals in the United States and up to 200,00 new cases per year [2]. Suboptimal candidates for revascularization include up to 15% of those who have ischemia or angina and are having cardiac catheterization [1]. A wide range of therapy approaches that focus on the pathophysiological patterns, effects, and underlying causes of disease are available. Comorbidities, advanced age, and poor coronary anatomy brought on by widespread disease or persistent complete occlusions, can be reasons for ineligibility for additional surgical revascularization [2]. Although the prognosis of refractory angina improved at a high cost of resources, annual mortality remains high at 3%-5% [1]. These results emphasize how imperative it is for these patients to get novel therapies [1]. 

Coronary microcirculation has recently been identified as a key pathophysiological mechanism in refractory angina [3]. Therapeutic angiogenesis has attracted continuous interest as a way to increase myocardial perfusion [2]. In vitro, a cluster of differentiation 34 positive (CD34+) stem cells stimulate the growth of new capillary networks, and in vivo, they incorporate into the regions of active angiogenesis in ischemic tissues [4]. Given that CD34+ stem cells can repair microcirculation, cell treatment employing autologous stem cells expressing CD34 offers a novel treatment option for patients with refractory angina [5]. The ability of a CD34+ stem cell to stimulate neo-angiogenesis and cardiomyocyte regeneration distinguishes it as a multipotent hematopoietic stem cell, also known as an endothelial progenitor cell. CD34+ stem cells are easily recruited from the bone marrow into peripheral circulation in response to ischemic tissue injury [6]. The role of progenitor cells in the presence of tissue injury and the precise nature of the precursors that affect repair are obvious questions to ask, given the evidence of the endothelial progenitor cell (EPC) contribution to the maintenance of vascular integrity provided by animals and human transplantation studies [7]. First, Losordo et al. [8] demonstrated the safety and beneficial efficacy of CD34+ stem cell treatment in patients with refractory angina [6]. The main benefits were a reduction in nitroglycerin use and the number of weekly angina attacks, as well as an improvement in exercise tolerance, Canadian Cardiovascular Society categorization, and quality of life [6]. Despite many effective therapies for angina, patients who remain symptomatic on maximally tolerated therapy create an unmet clinical need for new safe, and effective therapies. Thus, determining the utility of autologous CD34 + stem cell therapy for refractory angina is the main goal of this systematic review.

## Review

Methods

Searches in PubMed, PubMed Central (PMC), and Google Scholar yielded the relevant articles for this systematic review. As part of our systematic review, we followed preferred reporting items for systematic reviews and meta-analyses (PRISMA) as a reporting guideline [9]. In our search strategy, we used the search terms, as well as all possible synonyms. To find studies with potentially suitable content from the PubMed Databases, we used the Medical Subject Headings (MeSH) strategy.

Eligibility Criteria and Data Extraction

We include free full-text articles in our systematic review from the past 10 years (2012-2022) published in English, including clinical trials, meta-analyses, randomized controlled trials, reviews, and systematic reviews. As a result of a large number of articles on Google Scholar and PMC, only the first 300 articles from Google Scholar and the first 600 articles from PMC were reviewed. We imported and managed all references in Zotero (Reference Manager). All relevant data from eligible studies were extracted for duplicate, and discrepancies were resolved by discussion between reviewers. The field search is performed using keywords and Medical Subject Headings (MeSH) based on the database used (Table 1).

**Table 1:** Searching Through Databases Using Appropriate Filters for Conducting Field Searches PMC: PubMed central, CD34+: Cluster of differentiation 34 positive

Databases	Keywords	Search Strategy	Filters	Search Results
PubMed	CD34+ stem cell therapy OR Stem cell Transplantation Angina Pectoris OR Refractory Angina OR Coronary Microvascular Dysfunction OR Microvascular Angina OR Chest Pain OR Myocardial Ischemia	#1 CD34+ stem cell therapy OR stem cell transplantation OR (“Stem Cell Transplantation/adverse effects"[Majr] OR “Stem Cell Transplantation/organization and administration"[Majr] OR “Stem Cell Transplantation/pharmacology"[Majr] OR “Stem Cell Transplantation/therapeutic use"[Majr] OR “Stem Cell Transplantation/therapy"[Majr] ) #2 Angina pectoris OR refractory angina OR coronary microvascular dysfunction OR microvascular angina OR chest pain OR myocardial ischemia OR ( "Angina Pectoris/drug therapy"[Majr] OR "Angina Pectoris/immunology"[Majr] OR "Angina Pectoris/organization and administration"[Majr] OR "Angina Pectoris/prevention and control"[Majr] OR "Angina Pectoris/rehabilitation"[Majr] OR "Angina Pectoris/therapy"[Majr] ) OR ( "Microvascular Angina/drug therapy"[Majr] OR "Microvascular Angina/immunology"[Majr] OR "Microvascular Angina/prevention and control"[Majr] OR "Microvascular Angina/rehabilitation"[Majr] OR "Microvascular Angina/therapy"[Majr] ) OR ( "Myocardial Ischemia/drug therapy"[Majr] OR "Myocardial Ischemia/immunology"[Majr] OR "Myocardial Ischemia/organization and administration"[Majr] OR "Myocardial Ischemia/prevention and control"[Majr] OR "Myocardial Ischemia/rehabilitation"[Majr] OR "Myocardial Ischemia/therapy"[Majr] ) #1 AND #2 - 504	Free full text, Clinical Trial, Meta-Analysis, Randomized Controlled Trial, Review, Systematic Review, 10 years, English	270
PMC	CD34+ stem cell therapy OR Stem cell Transplantation Angina Pectoris OR Refractory Angina OR Coronary Microvascular Dysfunction OR Microvascular Angina OR Chest Pain OR Myocardial Ischemia	#1 CD34+ stem cell therapy OR stem cell transplantation OR (“Stem Cell Transplantation/adverse effects"[Majr] OR “Stem Cell Transplantation/organization and administration"[Majr] OR “Stem Cell Transplantation/pharmacology"[Majr] OR “Stem Cell Transplantation/therapeutic use"[Majr] OR "Stem Cell Transplantation/therapy"[Majr] ) #2 Angina pectoris OR refractory angina OR coronary microvascular dysfunction OR microvascular angina OR chest pain OR myocardial ischemia OR ( "Angina Pectoris/drug therapy"[Majr] OR "Angina Pectoris/immunology"[Majr] OR "Angina Pectoris/organization and administration"[Majr] OR "Angina Pectoris/prevention and control"[Majr] OR "Angina Pectoris/rehabilitation"[Majr] OR "Angina Pectoris/therapy"[Majr] ) OR ( "Microvascular Angina/drug therapy"[Majr] OR "Microvascular Angina/immunology"[Majr] OR "Microvascular Angina/prevention and control"[Majr] OR "Microvascular Angina/rehabilitation"[Majr] OR "Microvascular Angina/therapy"[Majr] ) OR ( "Myocardial Ischemia/drug therapy"[Majr] OR "Myocardial Ischemia/immunology"[Majr] OR "Myocardial Ischemia/organization and administration"[Majr] OR "Myocardial Ischemia/prevention and control"[Majr] OR "Myocardial Ischemia/rehabilitation"[Majr] OR "Myocardial Ischemia/therapy"[Majr] ) #1 AND #2 - 33180	Open access, last five years	14402
Google Scholar	CD34+ stem therapy cell, Angina	CD34+ stem cell therapy in angina - 24100	2012 - 2022	15900

Study Results and Bias Assessment

**Figure 1:**
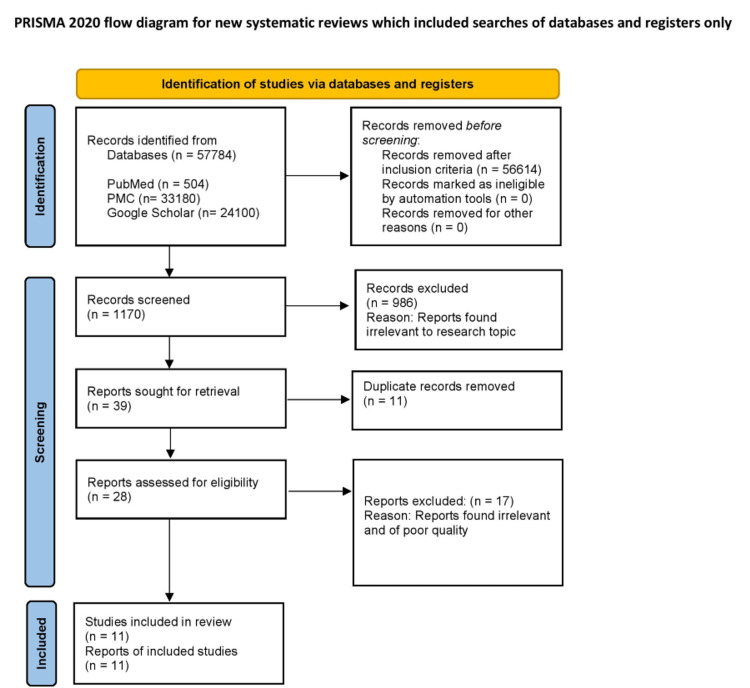
PRISMA 2020 Flow Diagram for Systematic Reviews PMC: PubMed central, PRISMA: Preferred reporting items for systematic reviews and meta-analyses

There were 57784 publications found in the initial search. Following the application of inclusion criteria, 56614 articles were excluded. After 1170 articles were screened for titles and abstracts, 986 were removed. Consequently, 39 papers with full text were retrieved, and 11 duplicate records were removed. In the end, 28 articles were reviewed, and 11 articles were included in the review (three randomized controlled trials, two meta-analyses, two non-randomized clinical trials, and four narrative reviews). A PRISMA flow diagram showing the screening process and study selection is presented in Figure 1 [9].

Risk of Bias

For the remaining full articles, we assessed quality assessment and risk of bias using the following tools: Randomized controlled trials were assessed with the Cochrane Collaboration’s Risk of Bias Tool (CCRBT) [10]; Non-randomized control trials were assessed with the Newcastle Ottawa Tool (NOS) [11]; narrative reviews were assessed with the scale for the assessment of narrative review articles 2 (SANRA 2) checklist [12]; meta-analysis was assessed with the assessment of multiple systematic reviews 2 (AMSTAR 2) checklist [13]. It was required that each assessment tool score of at least 70% be accepted (Table 2).

**Table 2:** Quality Assessment of Each Type of Study CCRBT: Cochrane Collaboration Risk of Bias Tool, NOS: Newcastle Ottawa Scale, AMSTAR 2: Assessment of multiple systematic reviews 2, SANRA 2: Scale for the assessment of narrative review articles 2, RCTs: Randomized controlled trials, RoB: Risk of bias, PICO: Patient/population, intervention, comparison, outcomes

Quality Assessment Tool	Type of Study	Items & their characteristics	Total score	Accepted score (>70%)	Accepted studies
CCRBT [10]	RCTs	Seven items: Random sequence generation and allocation concealment (selection bias), selective outcome reporting (reporting bias), other sources of bias, blinding of participants and personnel (performance bias), blinding of outcome assessment (detection bias), and incomplete outcome data (attrition bias). Bias is assessed as LOW RISK, HIGH RISK, or UNCLEAR.	7	5	Henry et al. [14], Povsic et al. [15], Johnson et al. [2]
NOS [11]	Non-Randomized Clinical Trials	Eight items: (1) Representativeness of the exposed cohort (2) Selection of the non-exposed cohort (3) Ascertainment of exposure (4) Demonstration that an outcome of interest was not present at the start of study (5) Comparability of cohorts based on the design or analysis* (6). Assessment of outcome (7) Was follow-up long enough for outcomes to occur (8) Adequacy of follow-up of cohorts. Scoring was done by placing a point on each category. Scored as 0, 1, 2. * Maximum of two points are allotted in this category.	9	7	Corban et al. [4], Henry et al. [16],
SANRA 2 [12]	Narrative Review	Six items: justification of the article’s importance to the readership, statement of concrete aims or formulation of questions, description of the literature search, referencing, scientific reason, and appropriate presentation of data. Scored as 0, 1, or 2.	12	9	Prasad et al. [17], Matta et al. [6], Rai et al. [5], Sietsema et al. [7]
AMSTAR 2 [13]	Meta-Analysis	Sixteen items: (1) Did the research questions and inclusion criteria for the review include the components of PICO? (2) Did the report of the review contain an explicit statement that the review methods were established prior to the conduct of the review, and did the report justify any significant deviations from the protocol? (3) Did the review authors explain their selection of the study designs for inclusion in the review? (4) Did the review authors use a comprehensive literature search strategy? (5) Did the review authors perform study selection in duplicate? (6) Did the review authors perform data extraction in duplicate? (7) Did the review authors provide a list of excluded studies and justify the exclusions? (8) Did the review authors describe the included studies in adequate detail? (9) Did the review authors use a satisfactory technique for assessing the risk of bias (RoB) in individual studies that were included in the review? (10) Did the review authors report on the sources of funding for the studies included in the review? (11) If meta-analysis was justified, did the review authors use appropriate methods for the statistical combination of results? (12) If a meta-analysis was performed did the review authors assess the potential impact of RoB in individual studies on the results of the meta-analysis or other evidence synthesis? (13) Did the review authors account for RoB in individual studies when interpreting/ discussing the results of the review? (14) Did the review authors provide a satisfactory explanation for and discussion of any heterogeneity observed in the results of the review? (15) If they performed quantitative synthesis, did the review authors carry out an adequate investigation of publication bias (small study bias) and discuss its likely impact on the results of the review? (16) Did the review authors report any potential sources of conflict of interest, including any funding they received for conducting the review? Scored as YES or NO. A partial Yes was considered as a point.	16	12	Li et al. [18], Henry et al. [1],

Results

The essential elements of clinical trials, reviews, and meta-analyses are outlined in Table 3.

**Table 3:** Key Characteristics of the Considered Studies CCS class III: Canadian Cardiovascular Society class three, CCS class IV angina: Canadian Cardiovascular Society class four angina, L-arginine: Levorotatory arginine, CBF: Coronary blood flow, SAQ: Seattle Angina Questionnaire, CFR: Coronary flow reserve, CMD: Coronary microvascular dysfunction, SF-36: Short form 36, MACE: Major adverse cardiac events, MI: Myocardial infarction, ACS: Acute coronary syndrome, QoL: Quality of life, PCI: Percutaneous coronary intervention, ETT: Exercise treadmill tests, TET: Total exercise time, CI: Confidence interval, OR: Odds ratio, RA: Refractory angina, CV: Cardiovascular, CVD: Cardiovascular disease, AF: Angina frequency, AMI: Acute myocardial infarction, INOCA: Ischemia with non-obstructive coronary arteries, RCT: Randomized controlled trial, SD: Standard deviation, CD34: Cluster of differentiation 34

Author and Year of Publication	Study Type	Patient Criteria	Outcome	Results	Conclusion
Corban et al, 2022 [4]	Open-label, single-center, single-arm, prospective clinical trial	Eligibility criteria: All patients had baseline CCS angina class III (n=seven; 35%) or IV (n=13; n=65%). Most patients were on statins and calcium channel blockers, and about half of the patients were on long-acting nitrates and L-arginine, n= 21 Mean age: 51.0±12.1 Years: one patient was excluded due to insufficient autologous CD34+ cell mobilization and low final cell purity. The 20 patients were recruited. Apart from one patient who missed her six-month follow-up appointment due to the COVID-19 pandemic, all 19 patients were evaluated at the six-months. In addition to that, another patient made the personal decision to complete the noninvasive but not invasive six-month follow-up.	Primary outcome: effect on coronary endothelial function at six months post-cell infusion, measured as the change in CBF and coronary artery diameter changes. Secondary outcome: Changes in CCS angina class, as-needed daily sublingual nitroglycerin use, individual SAQ domain scores, and SAQ summary score (overall metric of angina severity), and exercise time at six months	At six months: coronary blood flow increase (Mann-Whitney U test, P=0.035), with no significant interval change in coronary artery diameter percentage change (Mann-Whitney U test, P=0.25). Significant improvement in CCS angina class (P=0.00018) and decrease in as-needed sublingual nitroglycerin use per day (0.00047) no significant change in mean±SD treadmill exercise time (Wilcoxon signed-rank test, P=0.98).	Intracoronary injection of autologous CD34+ cells improved/normalized coronary microvascular endothelial function decreased the need for daily sublingual nitroglycerin, and enhanced anginal symptoms and quality of life.
Henry et al, 2022 [16]	Pilot Clinical Trial	Eligibility criteria: Men and women at least 18 years old, suffer from effort-induced angina, have three or more angina attacks per week, have no obstructive coronary artery disease (defined as CFR 2.5 to intracoronary adenosine on a clinically indicated angiogram within six months before consent), and have invasively confirmed endothelial independence from CMD (defined as CFR 2.5 to intracoronary adenosine at screening or within six months be 20 participants participated in a two-center experiment. All trial participants were expected to continue taking their regular medications.	Efficacy measures: Coronary flow reserve, angina frequency, Canadian Cardiovascular Society angina class, Seattle Angina Questionnaire, SF-36, and modified Bruce exercise treadmill test obtained at baseline and six months after treatment.	Improvement in Coronary flow reserve (P<0.005), Canadian Cardiovascular Society class (P<0.001), quality of life assessed by the Seattle Angina Questionnaire (P≤0.03, all scales), and SF-36 (P≤0.04, all scales). Angina frequency decreased (P<0.004). No cell-related serious adverse events.	After receiving an intracoronary infusion of CD34+ cells, patients with ischemia and nonobstructive coronary artery disease had higher coronary flow reserves, less severe angina, and better quality of life.
Henry et al, 2016 [14]	A phase II, prospective, double-blind, randomized, placebo-controlled clinical trial	Patients with class III-IV chronic refractory angina, despite adequate medical management and a planned 12-month follow-up ranging in age from 21 to 80, had an exercise restriction of between three and 10 minutes, at least seven angina events per week, and were not candidates for revascularization. A study was started with all patients (n=174), 167 patients were randomly assigned to undergo an intramyocardial injection of 1 × 10^5^ adult autologous CD34+ cells/kg, 5 × 10^5^ autologous CD34+ cells/kg, or placebo. Patients who completed 12 months of follow-up (n=156) were offered to participate in this continued 12- to 24-month follow-up study.	Safety endpoints: MACE [death, MI, ACS, hospitalization, worsening congestive heart failure, or stroke] were assessed at 15, 18, 21, and 24 months. Efficacy endpoints: angina frequency, CCS angina classification, SAQ, SF-36, and QoL were assessed at 18 and 24 months.	There were seven deaths during the 12- to 24-month period: four in the control group, one in the low-dose group (1 × 10^5^ cells/kg), and two in the high-dose group (5 × 10^5^ cells/kg). The relative risks for the first hospitalization for cardiac causes were 0.75 and 0.79 for the low- and high-dose groups, respectively, but did not reach statistical significance. Reduction in angina episodes per week at 12 months persisted to 24 months in both the low- and high-dose cell therapy groups. About QoL, angina stability score, SF-36 improved, although this did not meet statistical significance.	When CD34+ cells were injected intramyocardially into patients with CCS functional class III-IV angina who had failed conventional medical therapy and were not revascularization candidates, angina continued to improve two years later.
Johnson et al, 2020 [2]	Double-blinded, placebo-controlled randomized clinical trial	Class III–IV chronic refractory angina, according to the Canadian Cardiovascular Society, despite optimal medical management, and be ruled ineligible for further revascularization via PCI involving stenting or bypass surgery with at least seven angina attacks per week and restricted exercise tolerance. Three dosages of CD34+ cells—1 x 10^4^, 1 x 10^5^, or 5 x 10^5^ cells per kilogram—were given to patients at random, along with a placebo. For this study, a total of 37 patients who received autologous CD34+ cells were involved. 13.5% of patients were female, with a mean age of 57.9 ±7.5 years.	Cardiac-related emergency department visits and hospital admissions before and after injection of CD34+ cells or placebo, as well as associated hospital costs.	Visits in the year following CD34+ cell therapy: 50% reduction in hospital (P = .002), 73% reduction in coronary Procedures (P < .0001), 62% reduction in hospital costs (p= 0.03), Significant reduction in mortality (p= 0.02)	CD34+ cell therapy for patients with refractory angina is linked to better mortality, decreased hospital visits, and lower costs for cardiac procedures.
Povsic et al, 2016 [15]	A Randomized, double-blind, multicenter trial conducted at 45 centers in the United States	Eligibility criteria: between 18 and 80 years of age; had Canadian Cardiovascular Society class III or IV angina; an ejection fraction of 25%; reproducible (within 20%) exercise-limiting angina (between three and 10 minutes on two consecutive modified Bruce protocol exercise treadmill tests [ETT]); reported a minimum of seven angina episodes per week during a four-week screening period; were on maximally tolerated medical therapy, including b-blockers, calcium channel blockers, nitrates, and ranolazine; and had demonstrable ischemia on stress testing.	The primary efficacy endpoint: change from baseline in total exercise time (TET). The principal safety endpoint: is the incidence of MACE, including all-cause death, myocardial infarction, stroke, or cardiovascular hospitalization through 24 months. Secondary endpoints: change in TET at three and six months and change in angina frequency at three, six, and 12 months.	The difference in TET between patients treated with stem cells compared to placebo was 61.0 s (three months) (95% confidence interval (CI): -2.9 to 124.8; p ¼ 0.06), 46.2 s (six months) (95% CI: -28.0 to 120.4; p ¼ 0.22), and 36.6 s (12 months) (95% CI: -56.1 to 129.2; p ¼ 0.43); angina frequency was improved at six months (relative risk: 0.63, p ¼ 0.05). Major adverse cv incidents were 67.9% (in patients with the standard of care), 42.9% (in active control), and 46.0% [in patients receiving CD34+].	Due to the early termination, trial was an incomplete experiment; however, the results were consistent with observations from earlier phase studies.
Henry et al, 2018 [1],	A patient-level pooled meta-analysis of randomized double-blinded trials	The complete details of Phase I, Phase II ACT-34, and Phase III RENEW trials have been included. Patient characteristics: the median age of 63 years, Caucasian males account for a large proportion of participants, and most have cardiovascular risk factors, including diabetes in more than 50% of cases. The patients had also undergone prior coronary artery bypass graft surgery and/or percutaneous coronary intervention in nearly 90% of cases.	TET was assessed at the beginning and three months in Phase I, and at the beginning and three-, six-, and 12 months in ACT-34 and RENEW trials. ETT from ACT-34 and RENEW were interpreted and quantified by an independent core laboratory (Harvard University). Angina frequency was measured at the beginning, three- and six months in each study, and at 12 months in ACT-34 and RENEW trials. Side effects like death, MI, stroke, and CV hospitalizations were reported from six months in Phase I to 12 months in ACT-34 and 24 months in the ACT-34 extension and RENEW studies.	Statistically significant improvement in total exercise time, a 10%-20% reduction in the AF, but not significant statistically, although not significant, a clinically significant reduction in MACE (38.9% vs. 30.0%; P= 0.14) that was due to a reduction in all-cause death and CV hospital admission.	A combined analysis of three consecutive double-blinded clinical studies, including patients with Class III and IV RA, showed consistent and long-lasting improvements in exercise capacity, AF, and mortality with CD+34 stem cell therapy.
Li et al, 2013 [18]	A Meta-Analysis of Randomized Controlled Trials	The authors included five randomized controlled trials, with a total of 381 patients. Inclusion criteria: (1) a randomized controlled trial (RCT); (2) the patients with RA and not candidates for percutaneous coronary intervention or coronary artery bypass graft (3) the study involved autologous stem or progenitor cell therapy without restriction by cell type, dose, or administration route; and (4) it was an intention-to-treat analysis.	Net changes in exercise tolerance and angina frequency.	Significant improvement in exercise tolerance of 61.3 seconds (95% confidence interval [CI], 18.1- 104.4; P ¼ 0.005); an obvious reduction in angina frequency of 7.3 episodes per week (95% CI, -13.4 to -1.2; P ¼ 0.02) and decrease in MI risk, with an OR of 0.37 (95% CI, 0.14-0.95; P ¼ 0.04%). In death risk there was no difference detected (OR, 0.33; 95% CI, 0.08-1.39; P ¼ 0.13).	In the treatment of individuals with refractory angina, stem cell therapy seems to be safe and effective.
Prasad et al, 2020 [17]	Narrative Review	The authors performed a comprehensive search of several databases to identify preclinical and clinical studies using autologous CD34+ cell therapy for the treatment of CVD, including heart failures, such as dilated cardiomyopathy and ischemic cardiomyopathy, acute myocardial infarction (AMI), and refractory angina.			It is plausible that CD34+ cells could be a treatment option for patients with few options, such as those with coronary endothelial dysfunction and microvascular disease. However, more research is required to fully understand the potential role of CD34+ cells in treating symptomatic patients with non-obstructive coronary artery disease, microvascular disease, and endothelial dysfunction.
Matta et al, 2021 [6]	Narrative Review	The authors reviewed the potential utility of CD34+ cell transplantation in acute myocardial infarction, refractory angina, and ischemic heart failure.			Autologous CD34+ cell transplantation is safe and effective in treating AMI, refractory angina, and systolic heart failure, according to numerous clinical studies.
Rai et al, 2021 [5]	Narrative Review	The authors reviewed preclinical and clinical data leading to cell-based therapy for ischemic repair, focusing on microvascular repair in CMD patients with INOCA.			For INOCA patients with CMD, cell treatment employing autologous CD 34+ stem cells are a promising new therapeutic approach.
Sietsema et al, 2019 [7]	Narrative Review	In this review, the authors summarized the preclinical and clinical evidence from over 700 patients in clinical trials of CD34+ cell therapy for ischemic events such as limb ischemia, myocardial ischemia, and cerebral ischemia.			Patients who mobilize CD34+ cells efficiently have improved outcomes after ischemic events.

Discussion

Identification of CD34+ Cells as Endothelial Progenitors and their Mechanism of Action

The sialomucin transmembrane protein CD34 is found on the cell membrane [17]. Endothelial cells, hemopoietic stem cells, and vascular endothelial progenitor cells are the main cell types that express the human cell surface protein CD34 [17]. The CD34 antigen allowed the identification and isolation of circulating autologous EPC. This EPC can regenerate blood vessels in vitro and integrate into ischemic tissue with active angiogenesis in vivo [17]. In reaction to ischemic tissue damage, CD34+ cells are released into the peripheral blood circulation [6]. The high proliferation and differentiation capacities of these cells are critical in the recovery from myocardial damage [6]. The agents involved in the movement of CD34+ cells from the bone marrow to the peripheral circulation were discovered to include integrin antibodies, cysteine-rich angiogenic protein 61, stromal cell-derived factor 1 (SDF-1), and granulocyte colony-stimulating factor [19,20]. After migrating to the targeted area, CD34+ cells stimulate angiogenesis, neovascularization, and heart regeneration in two different ways [6]. First, smooth muscle cells and endothelial cells, which are the primary structural elements of interior vascular walls and result in vascular re-endothelialization, differentiate from CD34+ cells [6]. Second, they play a significant paracrine role in secreting substances that promote vasculogenesis, decrease the rate of cardiomyocyte and endothelial cell death, alter the extracellular matrix, and activate more progenitor cells [6]. They produce exosomes (membrane-bound nanovesicles), which play a significant role in their proangiogenic process [6]. The proangiogenic microribonucleic acids (microRNAs) that these exosomes transmit may increase stem cell activity and describe how CD34+ stem cell therapy has angiogenic and therapeutic benefits [6]. In humans, decreased levels of circulating CD34+ cells are linked to more severe coronary artery disease and worse outcomes following myocardial infarction, and higher mortality [1]. Autologous CD34+ cell treatment appears to improve endothelium-dependent/independent microvascular dysfunction, according to a few published data [6]. A considerable increase in coronary flow reserve has been found in earlier experiments [6].

Preclinical Research on CD34 Therapy for Ischemic Disease

After bench-top experiments, athymic mouse and rabbit models of hindlimb ischemia were given human CD34+ cells. To use human cells rather than rodent cells and preventing a host immunological response, athymic models were used. Analysis revealed that, in contrast to 1.6 ± 0.8% of tagged human CD34 cells, in 13.4 ± 5.7% of the capillary channel walls of athymic hindlimb ischemic mice, tagged human CD34+ cells were found to be incorporated. An ischemic limb is the only site where these cells are found. CD34+ stem cells were found to target ischemic tissues and physically integrate into vessels during these studies [5]. In a report published by Tsuji et al. [21], CD34+ cell therapy was explored for cerebral ischemia, showing the benefits of intravenous administration of human umbilical cord blood (hUCB)-CD34+ cells in a mouse model of neonatal stroke, including improved blood flow and reduced loss of ipsilateral hemispheric volume [7].

These studies were expanded to include myocardial ischemia in addition to peripheral ischemia. According to a study, a single systemic injection of human CD34+ cells enhanced capillary density and blood flow in the infarct zones by inducing angiogenesis and vasculogenesis. This was done after inducing an acute myocardial infarction (AMI). Furthermore, this research found that ischaemic mice with CD34+ stem cells therapy experienced a mean 22% (p<0.001) recovery in left ventricular ejection fraction (LVEF). Thus, it was determined that CD34+ cells improved the myocardial function in addition to increasing angiogenesis [5,7,22].

The research was also carried out in four cynomolgus monkeys that had autologous CD34+ stem cells injected into the pre-ischemic zone following the closure of the left anterior descending artery (LAD). These authors demonstrated that as compared to a group that got saline treatment, macaques who received intracardiac CD34+ cells displayed improvements in regional blood flow and fractional shortening [5,23]. This suggested that, in addition to direct integration, CD34+ stem cells may also function via recruiting angiogenic cytokines through paracrine signaling to cause angiogenesis [5]. Previous in vitro cell studies demonstrating the expression of several growth factors, cytokines, and chemokines linked to hematopoiesis and angiogenesis by CD34+ cells provide support for this mechanism [5].

Role of CD 34+ Stem Cell Therapy in Refractory Angina

Intracoronary CD34+ cell therapy for the treatment of coronary endothelial dysfunction in patients with angina and nonobstructive coronary arteries (IMPROvE-CED) trial was a prospective, open-label, single-center, single-arm study published in 2022 done by Corban et al. [4], showed that intracoronary autologous CD34+ cell therapy could enhance coronary microvascular endothelial function in Non-obstructive coronary artery disease (NOCAD) patients with invasive endothelial dysfunction and refractory angina despite maximum tolerated conventional medical treatment. Originally 21 patients were involved in the study. One patient was excluded due to insufficient autologous CD34+ cell mobilization and low final cell purity. The 20 patients were recruited. Apart from one patient who missed her six-month follow-up appointment due to the coronavirus disease (COVID-19) pandemic, all 19 patients were evaluated for six months. In addition to that, another patient made the personal decision to complete the noninvasive but not invasive six-month follow-up. Cells were collected by leukapheresis on day five after autologous CD34+ cell mobilization with daily subcutaneous Granulocyte-colony stimulating factor (GCSF) injections for five days. Isolated CD34+ cells were selectively infused into the patient's left anterior descending coronary artery (1×10^5^ cells/kg) in the cardiac catheterization laboratory. Primary and secondary outcomes were measured at one, three, and six months. Regarding primary outcomes, coronary blood flow increases (P=0.035) without a discernible change in the percentage of coronary artery diameter percentage change (P=0.25) at six months. Regarding secondary outcomes, significant improvement in Canadian Cardiovascular Society (CCS) angina class (P=0.00018) and a decrease in as-needed sublingual nitroglycerin use per day (0.00047) no significant change in mean ± standard deviation (SD) treadmill exercise time (Wilcoxon signed-rank test, P=0.98) at six months. No severe adverse events occurred during the study. With GCSF subcutaneous injections, mild-to-moderate adverse effects, such as pain in bone (n=15; 75% of cases) and nausea (n=13; 65% of cases), occurred. The study result suggests a potential mechanism and function for autologous CD34+ cell therapy in treating coronary endothelial dysfunction (CED) in people [4].

Henry et al. conducted an autologous CD34+ cell therapy trial (published in 2022) in two centers involving 20 participants with ischemia and NOCAD with persistent angina and coronary flow reserve ≤2.5 [16]. Granulocyte-colony stimulating factor 5 g/kg/day for five days was used to mobilize CD34+ cells. Cells were collected with leukapheresis. Isolated CD34+ cells in cell-function-enhancing media were given to participants in the intracoronary left anterior descending as a single dose. At baseline and six months after treatment, coronary flow reserve, angina frequency, Canadian Cardiovascular Society (CCS) angina class, Seattle Angina Questionnaire, short form 36 (SF-36), and modified Bruce exercise treadmill test was used as efficacy measures. At six months following treatment, the coronary flow reserve (CFR) increased from 2.08 ±0.32 at baseline to 2.68 ±0.79 (P=0.005); the average number of angina episodes per day reduced from 4.42±3.10 to 2.02±2.06 (P=0.004); the Canadian Cardiovascular Society class improved from an average of 3.20±0.95 to 2.05±1.18 (P<0.001); improvements in Seattle Angina Questionnaire (P≤0.03); SF-36 score improved from 29.71±7.44 to 36.03±11.11 (P=0.001); total exercise time increased from 624±217 seconds to 685±194 seconds (P=0.41). No adverse cell-related events occurred. The feasibility and safety of intracoronary administering CD34+ cells to patients with ischemia with non-obstructive coronary arteries (INOCA) with coronary microvascular dysfunction (CMD) and persistent angina are established by this trial [16].

In a randomized control study conducted by Johnson et al. (published in 2020) [2], the authors evaluated the effect of CD34+ cell therapy on cardiac-related hospital visits and expenses in comparison to the 12 months before stem cell injection. Effective measures were tallied in retrospect for patients involved at one site in one of three (phase I/IIa, phase II ACT-34, and phase III RENEW) double-blinded, placebo-controlled CD34+ trials as compared to placebo. A total of 56 individuals were randomized, with 37 receiving CD34+ cell treatment and 19 receiving a placebo. An average of 0.78 ± 1.90 cardiac-related hospital visits occurred in patients who were randomly assigned to CD34+ cell therapy compared to 1.57 ± 1.39 cardiovascular hospital visits 12 months before injection (P =.002). During the 12 months following the cell therapy injections, the coronary procedures for the cell therapy group were reduced by 73%. After injection, median variable costs for all cardiac hospital visits and procedures significantly decreased by 62% in the cell therapy group (P = 0.03). Since enrollment, there has been a considerably lower rate of mortality in the CD34+ group than in the placebo group. Nine deaths overall (24%) occurred in the CD34+ group against eight (47%) in the placebo group. In addition to the fact that it is the first cost study to have a control group, it is significant that this is the first trial to show that the CD34+ cell product reduces mortality in addition to healthcare expenditures and adverse events [2].

To assess the safety and effectiveness of autologous CD34+ stem cell treatment in refractory angina, Henry et al. conducted a larger phase II, double-blind, placebo-controlled trial (published in 2016) in which 174 patients with refractory angina were enrolled [14]. One hundred sixty seven patients were randomized to receive an intramyocardial injection of 1 × 10^5^ adult autologous CD34+cells/kg, 5 × 10^5^ autologous CD34+ cells/kg, or a placebo [14]. Up to 24 months of follow-up was conducted to assess safety (major adverse cardiac events (MACE) [death, myocardial infarction (MI), acute coronary syndrome, hospital admission, worsening congestive heart failure, or stroke]) and efficacy measures (angina frequency, CCS angina classification, and Seattle Angina Questionnaire (SAQ), short form-36 (SF-36), and quality of life) [14]. Out of the 156 trial participants that finished the 12-month follow-up, 148 participated in the two-year follow-up study, and 130 completed the 24-month follow-up efficacy study. When compared to the placebo group at six and 12 months, the low-dose treated individuals experienced a substantial decrease in angina frequency (p = 0.02, 0.035) and an increase in exercise tolerance testing (ETT) time (p = 0.014, 0.017). Patients who received both low- and high-dose CD34+ cells experienced a substantial decrease in angina frequency at 24 months (p = 0.03) [14]. Over 24 months, seven deaths occurred in the control group (12.5%), compared with one in the low-dose group (1.8%) and two in the high-dose group (3.6%) (p = 0.08) [14]. At 24 months, MACE occurred in control, low-dose, and high-dose patients, respectively, at rates of 33.9%, 21.8%, and 16.2% (p = 0.08) [14]. The study found a trend toward lower mortality in individuals with no other options and refractory angina who received autologous CD34+ cell treatment at two years, along with durable improvement in angina [14].

A randomized, double-blind, multicenter trial was conducted by Povsic et al. (published in 2016) to determine improvement in total exercise time (TET) and angina frequency in patients with refractory angina after autologous CD34+ cell administration [15]. A total of 112 patients were randomized to intramyocardial CD34+ administration and without any intervention as an open-label standard of care or intramuscular (IM) placebo injections as an active control. The authors observed improvement in TET at three months (p=0.06), at six months (p=0.22), at 12 months (p=0.43), and angina frequency at six months (p=0.02) in the CD34+ group. In light of strategic considerations, the sponsor terminated the trial. The trial was an incomplete experiment due to its early termination. On the other hand, the outcomes matched what had been observed in earlier phase trials [15].

In the review conducted by Rai et al. (published in 2021) and Sietsema et al. (published in 2019), various clinical trials regarding CD34+ therapy in refractory angina have been summarized [5,7]. The phase I double-blinded, placebo-controlled trial included 24 patients with CCS class III (three) or IV (four) angina who were receiving optimal medical care, and it showed initial safety, as well as clinical improvement in the CD34+, treated individuals [8]. Phase II trials conducted by Losardo et al. [24], and Henry et al. [14], showed improvement in angina frequency (AF) and total exercise time after CD34+ cell therapy. Phase III RENEW, a double-blind, placebo-controlled trial conducted by Povsic et al., was designed for food and drug administration (FDA) approval based on improvements in TET. The trial was unfortunately terminated early due to financial issues [15]. According to a meta-analysis done in 2018 of three randomized, double-blinded trials (n = 304): phase I and II, ACT-34 extension, and phase III RENEW, CD34+ therapy improved TET, AF, and MACE in patients with refractory angina. At two, six, and 12 months, auto-CD34+ patients experienced a relative angina frequency of 0.78 (95% Confidence Interval (CI) 0.63-0.98; P = 0.032), 0.66 (0.48-0.91; P = 0.012), and 0.58 (0.38-0.88; P = 0.011) compared to placebo patients. TET was improved by 46.6 s (after three months, 95% confidence interval (CI) 13.0 s-80.3 s; P = 0.007; 49.5 s (after months, 95% CI 9.3-89.7; P = 0.016); and 44.7 s (after 12 months, 9 5% CI -2.7 s-92.1 s; P = 0.065). At 24 months, autologous CD34 + cell treatment quantitatively reduced MACE (38.9% vs. 30.0; P = 0.14) and significantly decreased mortality (12.1% vs. 2.5%; P = 0.0025) [1]. A randomized, double-blinded, placebo-controlled trial of CD34+ cell treatment (FREEDOM) for patients with coronary microvascular dysfunction and refractory angina but no obstructive coronary artery disease was also summarized in this review. With 105 patients expected to receive CD34+ cell treatment in a 2:1 randomization, the trial's enrollment began in October 2020. Efficacy endpoints are changed in coronary flow reserve, angina frequency, CCS class, TET, and health-related quality of life. Safety outcome measures are adverse events, death, MACE, laboratory investigation, physical examination, and vital signs. The outcome of the ongoing Phase II FREEDOM trial will establish the efficacy of CD34 treatment for persistent anginal pain (NCT04614467) [5].

A meta-analysis and systematic review performed by Li et al. (published in 2013) demonstrated the safety and efficacy of stem cell treatment in refractory angina [18]. The authors concluded the result by analyzing five randomized clinical trials in this article. They found an improvement in exercise tolerance of 61.3 seconds (95% confidence interval [CI], 18.1-104.4; P ¼ 0.005; I 2 ¼ 58%); an obvious reduction in angina frequency of 7.3 episodes per week (95% CI, -13.4 to -1.2; P ¼ 0.02; I2 ¼ 93%); and lower risk of myocardial infarction, with an OR of 0.37 (95% CI, 0.14-0.95; P ¼ 0.04; I2 ¼ 0%) in patients receiving stem cell therapy compared to controls. For the risk of mortality, no change was seen (OR, 0.33; 95% CI, 0.08-1.39; P = 0.13; I2 = 20%). The study was found to be effective with the need for large-scale and longer follow-up studies [18].

Limitation

Our systematic analysis has some limitations because only three databases were used in our research to determine the minimal number of publications. Other databases were excluded. Only free full-text papers were used in our review. The number of patients in many of these trials continues to be small, and the patient sample is highly selected. Additionally, many of these studies lack randomization or blinding. While further research addressing the function of autologous CD34+ cells in larger, well-designed trials is done, the present findings have limitations and should be interpreted with caution.

## Conclusions

A systematic review concluded that CD34+ stem cell therapy can be a safe and effective option for patients who have angina refractory to conventional medical treatment and who cannot be revascularized. Clinical trials support the effectiveness of therapy in decreasing CFR, reducing angina frequency, improving quality of life and exercise capacity, as well as decreasing mortality and cardiac-related hospitalizations. With the randomized placebo-controlled FREEDOM trial, further investigation of the concept beyond this proof-of-concept study is underway. Future investigations should concentrate on large-scale, multicenter, randomized trials with extended follow-ups to address the issues surrounding stem cell therapy and offer sufficient proof of its clinical utility. Refractory angina management and overall prognosis may be revolutionized once this treatment is approved.

## References

[1] Henry TD, Losordo DW, Traverse JH (2018). Autologous CD34+ cell therapy improves exercise capacity, angina frequency and reduces mortality in no-option refractory angina: a patient-level pooled analysis of randomized double-blinded trials. Eur Heart J.

[2] Johnson GL, Henry TD, Povsic TJ (2020). CD34(+) cell therapy significantly reduces adverse cardiac events, health care expenditures, and mortality in patients with refractory angina. Stem Cells Transl Med.

[3] Velagapudi P, Turagam M, Kolte D (2019). Intramyocardial autologous CD34+ cell therapy for refractory angina: A meta-analysis of randomized controlled trials. Cardiovasc Revasc Med.

[4] Corban MT, Toya T, Albers D (2022). IMPROvE-CED trial: Intracoronary autologous CD34+ cell therapy for treatment of coronary endothelial dysfunction in patients with angina and nonobstructive coronary arteries. Circ Res.

[5] Rai B, Shukla J, Henry TD, Quesada O (2021). Angiogenic CD34 stem cell therapy in coronary microvascular repair: A systematic review. Cells.

[6] Matta A, Nader V, Galinier M, Roncalli J (2021). Transplantation of CD34+ cells for myocardial ischemia. World J Transplant.

[7] Sietsema WK, Kawamoto A, Takagi H, Losordo DW (2019). Autologous CD34+ Cell Therapy for Ischemic Tissue Repair. Circ J.

[8] Losordo DW, Schatz RA, White CJ (2007). Intramyocardial transplantation of autologous CD34+ stem cells for intractable angina: a phase I/IIa double-blind, randomized controlled trial. Circulation.

[9] Page MJ, McKenzie JE, Bossuyt PM (2021). The PRISMA 2020 statement: an updated guideline for reporting systematic reviews. BMJ.

[10] Higgins JP, Altman DG, Gøtzsche PC (2011). The Cochrane Collaboration's tool for assessing risk of bias in randomised trials. BMJ.

[11] Stang A (2010). Critical evaluation of the Newcastle-Ottawa scale for the assessment of the quality of nonrandomized studies in meta-analyses. Eur J Epidemiol.

[12] Baethge C, Goldbeck-Wood S, Mertens S (2019). SANRA-a scale for the quality assessment of narrative review articles. Res Integr Peer Rev.

[13] Shea BJ, Reeves BC, Wells G (2017). AMSTAR 2: a critical appraisal tool for systematic reviews that include randomised or non-randomised studies of healthcare interventions, or both. BMJ.

[14] Henry TD, Schaer GL, Traverse JH (2016). Autologous CD34+ cell therapy for refractory angina: 2-year outcomes from the ACT34-CMI study. Cell Transplant.

[15] Povsic TJ, Henry TD, Traverse JH (2016). The RENEW trial: Efficacy and safety of intramyocardial autologous CD34+ cell administration in patients with refractory angina. JACC Cardiovasc Interv.

[16] Henry TD, Bairey Merz CN, Wei J (2022). Autologous CD34+ stem cell therapy increases coronary flow reserve and reduces angina in patients with coronary microvascular dysfunction. Circ Cardiovasc Interv.

[17] Prasad M, Corban MT, Henry TD, Dietz AB, Lerman LO, Lerman A (2020). Promise of autologous CD34+ stem/progenitor cell therapy for treatment of cardiovascular disease. Cardiovasc Res.

[18] Li N, Yang YJ, Zhang Q, Jin C, Wang H, Qian HY (2013). Stem cell therapy is a promising tool for refractory angina: a meta-analysis of randomized controlled trials. Can J Cardiol.

[19] Qin G, Ii M, Silver M (2006). Functional disruption of alpha4 integrin mobilizes bone marrow-derived endothelial progenitors and augments ischemic neovascularization. J Exp Med.

[20] Grote K, Salguero G, Ballmaier M, Dangers M, Drexler H, Schieffer B (2007). The angiogenic factor CCN1 promotes adhesion and migration of circulating CD34+ progenitor cells: potential role in angiogenesis and endothelial regeneration. Blood.

[21] Tsuji M, Taguchi A, Ohshima M (2014). Effects of intravenous administration of umbilical cord blood CD34(+) cells in a mouse model of neonatal stroke. Neuroscience.

[22] Kocher AA, Schuster MD, Szabolcs MJ (2001). Neovascularization of ischemic myocardium by human bone-marrow-derived angioblasts prevents cardiomyocyte apoptosis, reduces remodeling and improves cardiac function. Nat Med.

[23] Yoshioka T, Ageyama N, Shibata H (2005). Repair of infarcted myocardium mediated by transplanted bone marrow-derived CD34+ stem cells in a nonhuman primate model. Stem Cells.

[24] Losordo DW, Henry TD, Davidson C (2011). Intramyocardial, autologous CD34+ cell therapy for refractory angina. Circ Res.

